# Effects of a forest therapy camp on cancer survivors’ stress, mood and natural killer cells in Korea

**DOI:** 10.7586/jkbns.24.011

**Published:** 2024-08-20

**Authors:** Young Ran Chae, Su Youn Park, So Yean Kang, Hyo Young Kang, Sun Hee Lee, Young Mi Jo, In Sun Cheon

**Affiliations:** 1 College of Nursing, Kangwon National University, Chuncheon, Korea; 2 Department of Nursing Science, Yeoju University, Yeojusi, Korea; 3 Department of Nursing, Daewon University College , Jecheon, Korea; 4 Department of Nursing, Seojeong University, Yangju, Korea; 5 Department of Nursing, Doowon Technical University, Anseong, Korea; 6 Nursing Department, Kangwon National University Hospital, Chuncheon, Korea; 7 Department of Nursing, Andong Science College, Andong, Korea

**Keywords:** Forests, Cancer survivors, Stress, psychological, Emotions, Killer cells, natural

## Abstract

**Purpose:**

This study investigated changes in psychological and physiological indices in cancer survivors who participated in a forest therapy camp in Korea.

**Methods:**

A total of 37 cancer survivors (19 and 18 in the experimental and control groups, respectively) participated in this study. Over a 2-night and 3-day period, the participants in the experimental group took part in a forest therapy camp that included activities such as gymnastics, walking, five-senses experiences, and meditation. Both groups completed self-report questionnaires that measured their stress levels and profile of mood states, both before and after the forest therapy camp. Blood samples were collected to measure the levels of cortisol, serotonin, and natural killer (NK) cells.

**Results:**

After the forest therapy camp, the experimental group exhibited reduced stress levels (*p* = .031) and a significant improvement in total mood disturbance (*p* = .047) when compared with the control group. The level of serotonin also significantly increased (*p* < .001). However, in contrast to the prediction, a significant increase in cortisol was noted in the experimental group relative to the control group (*p* = .016). Moreover, no significant difference in NK cells was noted between the two groups.

**Conclusion:**

Forest therapy can be easily applied to cancer survivors. The positive psychological effects of the forest therapy camp were confirmed by improvements in stress and mood states and the increased level of serotonin in forest therapy camp participants. However, there is a need for a follow-up evaluation of cortisol and NK cells due to the absence of significant between-group differences.

## INTRODUCTION

Cancer remains the leading cause of death worldwide, while at the same time more adults than ever are surviving cancer [1]. The number of cancer patients in South Korea has increased rapidly over the past two decades. According to the 2021 National Cancer Registration Statistics, if South Korean citizens live to their life expectancy of 83.6 years, the probability of developing cancer is 38.1% [2]. While cancer appears to pose one of the most serious threats to human life, continuous advancements in healthcare technology have steadily increased the 5-year survival rate of patients in South Korea. The 5-year relative survival rate for cancer patients diagnosed in the last five years (2017-2021) is estimated to be 72.1% [2].

Cancer survivors experience a spectrum of difficulties between the diagnosis and treatment period, as well as post recovery. Mullan (1985) classified the stages of cancer and its treatment process into three distinct phases based on the changes in the state of the disease and treatment process: the acute survival phase, where the patient is actively undergoing treatment following a cancer diagnosis; the extended survival phase, where the patient receives follow-up care after the acute treatment; and the permanent survival phase, which occurs after the patient is considered cured. It is essential that they are provided with suitable care at each step because their needs vary during each period [3-5]. Notably, in particular, during the extended survival phase, individuals may experience physical and mental challenges such as stress, depression, and reduced physical stamina, in addition to the fear of recurrence [6].

Nature is beneficial for cancer survivors while they experience cancer diagnosis and treatment. Nature-based interventions have shown promising effects on overall health and well-being in the general population [1]. Forest therapy, one of the nature-based interventions, is proposed as a health promotion activity for the psychological, physical, and spiritual resilience of the subjects through various environment [7]. According to the Act on Forest Culture and Recreation, forest healing is defined as an activity that enhances the immune system and promotes health by utilizing various elements of nature, such as fragrances and landscapes [8]. Forest therapy is the principle of planning and developing programs using various forest environmental elements such as plants, water and nature sounds, and maximizing healing effects according to the intervention of forest therapy instructors [8]. Some related studies have noted improvements in the stress [9-12] and mood [11,12] of patients undergoing forest therapy. Forest therapy is also considered to relieve tension through the control of balance in the autonomic nervous system [13] and boost immune functions by relieving stress [14,15]. In spite of these positive effects only a few have investigated its effects on cancer survivors, for example, on quality of sleep in gastrointestinal cancer patients [16] and on depression and resilience in community-dwelling cancer patients [17]. This implies the need for investigating the potential contribution of forest therapy in maintaining the health of cancer survivors. Measuring cortisol, serotonin, and natural killer (NK) cells in cancer survivors is important for objectively assessing stress levels and mood states, as well as understanding immune function to monitor overall health. This enables the provision of tailored support during the treatment and recovery process. Additionally, it helps scientifically verify the effectiveness of nature-based interventions like forest therapy. Accordingly, this study aims to ascertain the effects of a forest therapy program on psychological indicators such as stress and mood states and physiological indicators such as cortisol, serotonin, and NK cells of patients who have completed adjuvant therapy with cancer.

## METHODS

### 1. Participants

The participants were individuals registered in the Cancer Survivor Integrated Support Center in Chuncheon city, South Korea. They had completed adjuvant therapy following cancer diagnosis. The experiment was approved by the institutional review board at Kangwon National University (KWNUIRB-2021-05-012). The inclusion criteria for this study were cancer patients aged 19 or above who had completed primary treatment to achieve complete remission. Participants were required to have no cognitive impairment or mental disorder, possess the ability to understand and respond to the questionnaire, and have no sensory dysfunction or lower extremity impairment, ensuring they could walk without assistance. Comprehensive explanations were provided regarding the study's rationale, purpose, invasive treatments, procedures, and duration. Only those who voluntarily agreed to participate and provided signed consent were included in the study.

**Figure 1.**
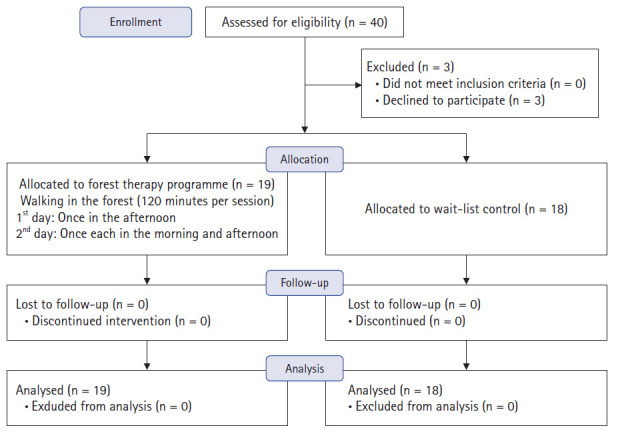
Flow diagram of study participants.

The sample size was determined using the G*power program. With an effect size of 0.8, a significance level of .05, and a power of 0.8, the required number of participants for each group was found to be 21 [18]. Twenty participants in each of the experimental and control groups agreed to participate in the study. However, one and two participants from the experimental and control groups, respectively, decided to withdraw from it. Finally, a total of 37 participants (19 and 18 from the experimental and control groups, respectively) participated in this study (Figure 1).

### 2. Forest therapy camp

The experiment was conducted between May 18 and May 20, 2022. The therapy sessions were held at the foot of Mt. Sobaeksan in Yeongju-si, Gyeongsangbuk-do, South Korea, in the national recreational forest enriched with the forest resources of the Baekdudaegan range. Under the guidance of a forest therapist, the experimental group performed activities aimed at monitoring the forest ecosystem and directly exploring the outdoor forest during the two-night and three-day period.

**Figure 2.**
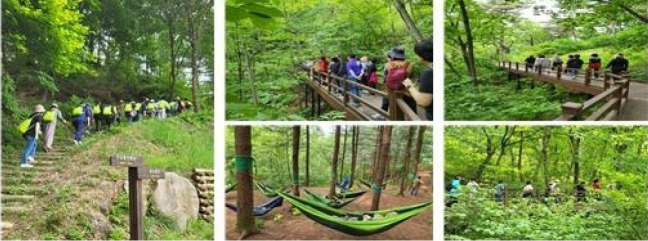
Image of forest therapy experiment.

A forest program largely encompasses six main therapeutic elements: plants, water, weather, dietary style, exercise, and psychotherapy [8]. The camp conducted a program that included these six elements. The forest therapy program involved three activity sessions (approximately 120 minutes per session): one session in the afternoon of the first day after the participants reached the location, and one session each in the morning and afternoon of the second day. On the first day, the program consisted of a slow walk through the forest combined with breathing relaxation exercises. After dinner, participants were encouraged to independently engage in light activities such as walking. On the second day, the morning included walking on the deck road in the forest, while the afternoon was dedicated to hammock meditation in a pine forest during a walk. On the third day, participants took a stroll near a stream in the forest (Figure 2). The control group was instructed to maintain their usual daily activities and refrain from participating in any forest activities during this period.

### 3. Instruments

Participants’ general characteristics, such as gender, age, marital status, diagnosed cancer type, experience of forest therapy, and normal level of exercise, were investigated.

#### 1) Stress

Stress was evaluated using a restructured Korean version of the Psychosocial Well-being Index- Short Form (PWI-SF), based on the general health questionnaire of Goldenberg [19]. The PWI-SF contained 18 questions. Each question was rated on a 4-point scale, with 0 indicating “Always true,” 1 indicating “Mostly true,” 2 indicating “Sometimes true,” and 3 indicating “Not at all true.” Negative questions were reverse coded. Lower total scores indicated lower levels of stress. The participants were classified as healthy if the PWI-SF score was ≤8, as a potential risk group if the score was between 9 and 26, and as a high risk group if the score was ≥ 27. The Cronbach’s ⍺ was .92 at the time of tool development and .84 in this study.

The level of cortisol in serum samples was evaluated. For serum cortisol, three milliliters of whole blood were collected and centrifuged at 7,000 rpm at room temperature for seven minutes. The sample was placed in a refrigerated case and sent to the EONE Laboratories for analysis via chemiluminescent enzyme immunoassay. Higher levels of cortisol indicate higher levels of stress.

#### 2) Mood states

Mood was evaluated using the Korean version of Profile of Mood States-Brief, a brief form of the questionnaire of McNair translated into Korean [20]. The tool contained 30 questions, with five questions each on tension, depression, anger, vigor, fatigue, and confusion. Each question was rated on a 5-point scale, with 0 indicating “Strongly disagree” and 4 indicating “Strongly agree.” The scores for the five subdomains of tension, depression, anger, fatigue, and confusion are summed, and then the score for the vigor subdomain is subtracted to calculate the Total Mood Disturbance (TMD) score. A higher TMD score indicates a worse mood state. For the mood state assessment, participants were instructed to quickly check the number corresponding to their initial emotional state as soon as it came to mind. The TMD has been primarily used to investigate the relationship between forest therapy and mood states [7,15,21]. The Cronbach’s ⍺ was .85 at the time of tool development [20] and .85 in this study.

Serotonin is a neurotransmitter that maintains a calm emotional state by suppressing stimulatory hormones such as dopamine in order to prevent overexcitement or anxiety. To measure the level of serotonin, three milliliters of whole blood were collected from each participant in an eight-hour fasting state between 7 a.m.–8 a.m., centrifuged at 7,000 rpm for seven minutes; the samples were then placed in a refrigerated case to be sent to EONE Laboratories for analysis via high-performance liquid chromatography. Serotonin is associated with depression and anxiety [22] a low level of serotonin results in a variety of problems, such as anxiety and depression [23]. In this study, higher serotonin levels indicated more comfort and less anxiety.

#### 3) NK cells

NK cells (CD16 + CD56) levels were measured using flow cytometry. NK cells constitute the human body’s main defense system of innate immunity along with T lymphocytes and B lymphocytes. To this end, 3 mL of blood was collected in ethylenediaminetetraacetic acid tubes and sent to the EONE Laboratories.

### 4. Data collection

The pre-test measurements for the experimental and control groups were performed at the Cancer Survivor Integrated Support Center at Kangwon National University Hospital. First, blood samples were collected. Second, the participants were guided to self-report their stress and mood states using a structured questionnaire. The blood samples were collected between 7 a.m.–8 a.m. when the participants were in an eight-hour fasting state. Three milliliters of whole blood were collected and stored in a refrigerated case to be transferred to the EONE Laboratories for the analysis of cortisol, serotonin, and NK cell levels.

Post-test measurements on identical indicators were performed at the end of the forest therapy intervention, in the same order as the pre-test measurements were taken. The experimental group's measurements were taken at the forest lodge, and the control group's measurements were taken at the same center of the university hospital where the pre-test measurements were taken.

### 5. Statistical analysis

Data analysis was conducted using SPSS/WIN 21.0 (IBM Corp., Armonk, NY, USA). To analyze the general characteristics of the participants and the disease-related characteristics, frequency and percentage or mean and standard deviation were estimated. The χ²-test or Fisher’s exact test were performed to test the homogeneity of the general characteristics of the experimental and control groups. To evaluate the effectiveness of the program, an independent t-test was performed, and in the case where normality was not satisfied, Wilcoxon rank sum test was conducted.

## RESULTS

### 1. General characteristics of the participants

The mean ages of the experimental and control groups were 58.26 (± 6.69) and 54.83 (± 6.15), respectively. The proportion of women was higher in both groups. The two groups were homogeneous in terms of gender, age, and marital status, based on the lack of significant variations. The observed heterogeneity in occupations is likely attributable to the convenience sampling of participants. Individuals with jobs were restricted from participating in the camp because they had to participate in a 3-day, 2-night forest therapy camp. The two groups were also found to be homogeneous in terms of sharing previous experiences in participating in the forest therapy program, forest walks, and regular exercises (Table 1).

Regarding cancer type, breast cancer was the most common in the experimental group with 14 patients (73.7%). Three of the 14 participants had a combination of breast cancer and another cancer (thyroid/fallopian tube/renal); One participant had been diagnosed with thyroid cancer and ovarian cancer with breast cancer. The remaining five participants had been each diagnosed with liver cancer (one participant, 5.3%), hematologic malignancy (5.3%), thyroid cancer (5.3%), ovarian cancer (5.3%), and endometrial cancer (5.3%).

Among the 18 participants in the control group, 13 had been diagnosed with breast cancer (72.2%); two of these participants had also been diagnosed with colon cancer and thyroid cancer each, and one of them had been diagnosed with renal cancer. The remaining five participants had been each diagnosed with thyroid cancer (one participant, 5.6%), liver cancer (one participant, 5.6%), endometerial cancer (one participant, 5.6%), lymphoma (one participant, 5.6%) and colorectal cancer (one participant, 5.6%).

### 2. Effects of the forest therapy on stress, mood states and NK cells

The stress score in the experimental group decreased from 1.08 (± 0.38) before the forest therapy intervention to 0.69 (± 0.45) after the intervention, while its decrease in the control group, from 1.75 (± 0.34) to 1.72 (± 0.34) was negligible. This indicated a significant between-group variation (t = -2.45, *p* = .031). In the mood scale, the experimental group showed significant decreases in tension scores from 0.81 (± 0.63) to 0.46 (± 0.56) (*p* = .042), depression scores from 0.75 (± 0.74) to 0.43 (± 0.06) (*p* = .046), fatigue scores from 0.99 (± 0.63) to 0.45 (± 0.61) (*p* = .010), and TMD scores from 2.37 (± 3.04) to 0.46 (± 2.78) (*p* = .047).

The cortisol level in the experimental group increased from 11.40 (± 4.27) before the therapy to 14.45 (± 3.32) after the therapy, while the cortisol level in the control group decreased from 11.61 (± 3.41) to 11.42 (± 4.27) (*p* = .016). The serotonin level increased in the experimental group but decreased in the control group after the therapy, displaying a significant variation (t = 4.13, *p* < .001). The level of NK cells displayed an insignificant between-group variation (Z = -1.13, *p* = .261) (Table 2).

## DISCUSSION

This study aims to ascertain the effects of the forest therapy camp on the psychological and physiological indicators including stress scores, mood scores, cortisol levels, serotonin levels, and NK cell counts in cancer survivors. In addition, the applicability of the forest therapy camp for cancer survivors was verified. All participants who participated in the camp did not complain of physical or mental difficulties in participating in the program and expressed satisfaction with the program.

Cancer survivors are reported to experience more stress and depression compared to the general population. They face psychological and emotional challenges during cancer diagnosis, treatment, and the recovery process. Stress and depression can severely impact the quality of life of cancer survivors, making it crucial to manage these psychological issues effectively [24]. Forest therapy in our study was found to reduce stress scores among cancer survivors. Although the study was not conducted on a similar group, this is consistent with a previous study on the remedial effect of forest therapy on the stress scores of local residents experiencing post-traumatic stress following a forest fire [25]. This is also in line with a study analyzing the effects of a forest therapy-based stress management program on middle-aged females [21]. Because it is important to understand and effectively manage mental health problems such as stress, anxiety, and depression experienced by cancer survivors [26], forest therapy is considered applicable to stress management in cancer survivors. Stress management has a significant impact on lifestyle changes and health outcomes for cancer survivors [27].

In this study, forest therapy had a significant effect on mood states, including depression and fatigue, and serotonin levels in cancer survivors. The result was similar to that of previous studies on the positive effects of forest activities in alleviating depression and fatigue [11,28,29]. Serotonin is a neurotransmitter influenced by daytime exposure to sunlight and physical activity. The participation in the forest therapy camp is presumed to have increased the time of sunlight exposure and physical activity, resulting in a significant increase in participants’ serotonin levels after the intervention. A study on middle-aged females found that forest therapy significantly increased participants’ serotonin levels after the intervention, from 147 ng/mL to 156 ng/mL [23], higher than those recorded in this study. Serotonin is associated with emotions such as depression and anxiety [22]. As cancer survivors experience depression, anxiety, and stress [30-33], it is presumed that the study participants’ levels of serotonin were inevitably slightly lower than normal. This finding is particularly important as serotonin is a key neurotransmitter involved in mood regulation. The increase in serotonin levels suggests that forest therapy may facilitate improved mental health through biochemical pathways [34].

Activities in a forest environment can reduce the level of cortisol among adults [35]. This was confirmed in two studies; the first was on middle-aged females (≥ 40 years) who participated in a single session of forest experience for approximately five hours [28], while the second was on male undergraduates [10]. In both cases, the level of cortisol decreased. The studies suggested that phytoncides in forest air promote defense mechanisms in the body, lowering the level of the stress hormone, cortisol [36]. The varying environmental factors of the forest were shown to increase participants’ psychological and physical stabilities while the activities involving both walking and meditating in a forest environment reduced their stress hormones [23]. However, despite the lower level of self-reported stress, the level of cortisol was found to increase in this study, as opposed to that observed in previous studies [10,11,28,35], which resulted in decreased cortisol level. This counterintuitive result might reflect a complex physiological adaptation process to the forest environment, warranting further investigation into the acute and chronic effects of forest therapy on the hypothalamic-pituitary-adrenal axis [37]. Because the second measurement was done at the forest lodge, the increase in cortisol levels observed in our study may be attributed to an initial acute stress response due to unfamiliarity with the environment or physical activity associated with walking in the forest. This initial increase does not necessarily indicate negative stress but could be a part of a positive physiological adaptation process to a new and beneficial stimulus. These findings underscore the need for more comprehensive studies to understand the underlying mechanisms and long-term effects of forest therapy on cortisol and overall stress physiology.

The level of NK cells, which play a crucial role in the body's immune response, did not show a significant between-group variation. Generally, the proportion of NK cells is about 15% of lymphocyte [38], but the participants in this study also showed a proportion within the normal range, so there does not seem to be a significant increase. This is probably because the participants in this study were cancer survivors who had completed treatment. Therefore, there is a need to explore the effects of forest therapy on NK cells in participants undergoing treatments.

Cancer survivors experience a considerable level of stress during the treatment and recovery processes [30,31], which can increase their levels of depression [32,33] and negatively affect their quality of life [39]. Additionally, the participants in this study were aged 40 or above and are experiencing midlife transitions. Middle-aged women may experience various menopausal symptoms, ranging from physical aging to psychological withdrawal, depression, fatigue, and insomnia [40]. Therefore, an intervention is required to help cancer survivors recover fully from treatment and maintain their health. Forest therapy has good efficacy in mitigating the negative consequences and sequela of cancer treatment and survivorship [1].

The limitation of this study is that forest therapy camp was conducted for only a short two-day and three-night period. To verify the clinical effectiveness of the forest therapy among cancer survivors, an extended study with a continuous program and long-term follow-up monitoring should be conducted. For this, a forest therapy program involving activities more closely related to daily life should be developed.

## CONCLUSION

In conclusion, the study demonstrates that forest therapy has significant positive effects on stress reduction and mood enhancement, with notable changes in serotonin levels. The increase in cortisol levels in the experimental group, however, suggests a need for more comprehensive studies to understand the underlying mechanisms. Despite the lack of significant change in NK cell counts, the overall findings support the integration of forest therapy into mental health and wellness programs. Future research should explore the long-term effects and optimal durations of forest therapy to fully harness its benefits.

## Figures and Tables

**Table 1.** Characteristics of Study Participants and Homogeneity Test (N = 37)

Characteristics	Categories	Exp.(n = 19)	Cont.(n = 18)	χ^2^	*p*
Age (yr)		58.26 ± 6.69	54.83 ± 6.15		
	40–49	2 (10.5)	5 (27.8)		.116^†^
	50–59	7 (36.8)	10 (55.6)		
	60–65	10 (52.7)	3 (16.6)		
Gender	Men	1 (5.3)	1 (5.6)		1.000^†^
	Women	18 (94.7)	17 (94.4)		
Marital status	Married	12 (63.2)	16 (88.9)		.669^†^
	Single/divorced/bereaved	7 (36.8)	2 (11.1)		
Occupation	Yes	3 (15.8)	13 (72.2)	11.99	.001
	No	16 (84.2)	5 (27.8)		
Type of cancer	Breast	14 (73.5)	13 (72.2)	0.27	.603
	Thyroid	1 (5.3)	1 (5.6)		
	Liver	1 (5.3)	1 (5.6)		
	Hematologic	1 (5.3)	-		
	Ovarian	1 (5.3)	-		
	Endometrial	1 (5.3)	1 (5.6)		
	Lymphoma	-	1 (5.6)		
	Colorectal	-	1 (5.6)		
Past forest therapy participation	Yes	1 (5.3)	1 (5.6)		1.000^†^
	No	18 (94.7)	17 (94.4)		
Forest walking	Yes	13 (68.4)	11 (61.1)	0.22	.642
	No	6 (31.6)	7 (38.9)		
Regular exercise	Yes	17 (89.5)	16 (88.9)		1.000^†^
	No	2 (10.5)	2 (11.1)		

Values are presented as the mean ± standard deviation or n (%). Exp. = Experimental group; Cont. = Control group.

† Fisher exact test.

**Table 2.** Differences in Stress, Mood, Cortisol, Serotonin and NK Cells Between the Control and Experimental Groups: Effect of Forest Therapy (N = 37)

Variables		Group	Baseline	After	Difference	t/Z	*p*
Stress		Exp. (n = 19)	1.08 ± 0.38	0.69 ± 0.45	-0.39 ± 0.42	-2.45	.031
		Cont. (n = 18)	1.75 ± 0.34	1.72 ± 0.34	-0.03 ± 0.50		
Mood	Tension	Exp .	0.81 ± 0.63	0.46 ± 0.56	-0.35 ± 0.55	-2.12	.042
		Cont.	0.69 ± 0.49	0.66 ± 0.51	-0.03 ± 0.31		
	Depression	Exp.	0.75 ± 0.74	0.43 ± 0.06	-0.32 ± 0.57	-2.07	.046
		Cont.	0.47 ± 0.49	0.49 ± 0.60	0.02 ± 0.41		
	Anger	Exp.	0.49 ± 0.58	0.29 ± 0.48	-0.20 ± 0.45	-0.94	.356
		Cont.	0.47 ± 0.43	0.39 ± 0.44	-0.08 ± 0.34		
	Vigor	Exp.	1.72 ± 0.89	1.95 ± 1.15	0.23 ± 1.15	0.33	.741
		Cont.	2.00 ± 0.59	2.12 ± 0.97	0.12 ± 0.81		
	Fatigue	Exp.	0.99 ± 0.63	0.45 ± 0.61	-0.54 ± 0.60	-2.72	.010
		Cont.	0.63 ± 0.57	0.69 ± 0.64	0.06 ± 0.73		
	Confusion	Exp.	1.04 ± 0.61	0.77 ± 0.51	-0.27 ± 0.64	-1.04	.305
		Cont.	1.09 ± 0.34	1.01 ± 0.37	-0.08 ± 0.49		
	Total mood disturbance	Exp.	2.37 ± 3.04	0.46 ± 2.78	-1.91 ± 2.69	-2.06	.047
		Cont.	1.13 ± 2.19	1.11 ± 2.86	-0.02 ± 0.27		
Cortisol (μg/dL)		Exp.	11.40 ± 4.27	14.45 ± 3.32	3.05 ± 4.21	2.52	.016
		Cont.	11.61 ± 3.41	11.42 ± 4.27	-0.19 ± 3.57		
Serotonin (ng/mL)		Exp.	109.72 ± 57.06	121.63 ± 67.13	11.91 ± 27.15	4.13	<.001
		Cont.	114.76 ± 55.58	93.05 ± 43.68	-21.71 ± 21.95		
NK-cells (%)		Exp.	14.28 ± 5.06	14.66 ± 7.44	0.38 ± 4.98	-1.13^†^	.261
		Cont.	18.53 ± 6.44	19.71 ± 6.76	1.18 ± 3.75		

Values are presented as the mean ± standard deviation. Exp. = Experimental group; Cont. = Control group; NK = Natural killer.

† Wilcoxon rank sum test.

## Data Availability

The data that support the findings of this study are available from the corresponding author upon reasonable request.
